# Increased Immunosignals of Collagen IV and Fibronectin Indicate Ischemic Consequences for the Neurovascular Matrix Adhesion Zone in Various Animal Models and Human Stroke Tissue

**DOI:** 10.3389/fphys.2020.575598

**Published:** 2020-10-26

**Authors:** Dominik Michalski, Emma Spielvogel, Joana Puchta, Willi Reimann, Henryk Barthel, Björn Nitzsche, Bianca Mages, Carsten Jäger, Henrik Martens, Anja K. E. Horn, Stefan Schob, Wolfgang Härtig

**Affiliations:** ^1^Department of Neurology, University of Leipzig, Leipzig, Germany; ^2^Paul Flechsig Institute for Brain Research, University of Leipzig, Leipzig, Germany; ^3^Department of Neuroradiology, University of Leipzig, Leipzig, Germany; ^4^Department of Nuclear Medicine, University of Leipzig, Leipzig, Germany; ^5^Institute of Anatomy, Histology and Embryology, Faculty of Veterinary Medicine, University of Leipzig, Leipzig, Germany; ^6^Institute of Anatomy, University of Leipzig, Leipzig, Germany; ^7^Synaptic Systems GmbH, Göttingen, Germany; ^8^Institute of Anatomy and Cell Biology I and German Center for Vertigo and Balance Disorders, Ludwig-Maximilians-University, Munich, Germany

**Keywords:** stroke, fibronectin, collagen IV, basement membrane, blood-brain barrier, extracellular matrix

## Abstract

Ischemic stroke causes cellular alterations in the “neurovascular unit” (NVU) comprising neurons, glia, and the vasculature, and affects the blood-brain barrier (BBB) with adjacent extracellular matrix (ECM). Limited data are available for the zone between the NVU and ECM that has not yet considered for neuroprotective approaches. This study describes ischemia-induced alterations for two main components of the neurovascular matrix adhesion zone (NMZ), i.e., collagen IV as basement membrane constituent and fibronectin as crucial part of the ECM, in conjunction with traditional NVU elements. For spatio-temporal characterization of these structures, multiple immunofluorescence labeling was applied to tissues affected by focal cerebral ischemia using a filament-based model in mice (4, 24, and 72 h of ischemia), a thromboembolic model in rats (24 h of ischemia), a coagulation-based model in sheep (2 weeks of ischemia), and human autoptic stroke tissue (3 weeks of ischemia). An increased fibronectin immunofluorescence signal demarcated ischemia-affected areas in mice, along with an increased collagen IV signal and BBB impairment indicated by serum albumin extravasation. Quantifications revealed a region-specific pattern with highest collagen IV and fibronectin intensities in most severely affected neocortical areas, followed by a gradual decline toward the border zone and non-affected regions. Comparing 4 and 24 h of ischemia, the subcortical fibronectin signal increased significantly over time, whereas neocortical areas displayed only a gradual increase. Qualitative analyses confirmed increased fibronectin and collagen IV signals in ischemic areas from all tissues and time points investigated. While the increased collagen IV signal was restricted to vessels, fibronectin appeared diffusely arranged in the parenchyma with focal accumulations associated to the vasculature. Integrin α_5_ appeared enriched in the vicinity of fibronectin and vascular elements, while most of the non-vascular NVU elements showed complementary staining patterns referring to fibronectin. This spatio-temporal characterization of ischemia-related alterations of collagen IV and fibronectin in various stroke models and human autoptic tissue shows that ischemic consequences are not limited to traditional NVU components and the ECM, but also involve the NMZ. Future research should explore more components and the pathophysiological properties of the NMZ as a possible target for novel neuroprotective approaches.

## Introduction

Despite enormous efforts concerning a more detailed understanding of pathophysiological mechanisms, ischemic stroke often leads to long-term disability and is still ranging among the three most common causes of death worldwide (Benjamin et al., 2018; Campbell et al., 2019). On the cellular level, excitotoxicity, apoptosis, and inflammation were identified as mechanisms contributing to the ischemia-related tissue damage in a complex time‐ and region-dependent manner (Dirnagl et al., 1999). Notwithstanding the emerging knowledge on stroke pathophysiology, the development of neuroprotective approaches is rather challenging (Young et al., 2007; Endres et al., 2008). Especially in the era of modern techniques for the recanalization of occluded brain vessels, i.e., intravenous thrombolysis (Hacke et al., 2018) and endovascular treatment (Goyal et al., 2016), additional neuroprotective approaches are needed to facilitate neuronal survival and to reduce bleeding complications.

More than 10 years ago, a group of regionally and functionally associated cells were termed as the “neurovascular unit” (NVU) in order to learn more than their roles and interplay during stroke evolution. Thus, the NVU consists of neurons, astrocytes, oligodendrocytes, microglia, the vasculature with its endothelium, and other cell types (Lo et al., 2003; del Zoppo, 2009, 2010; Iadecola, 2017). Over time, multiple ischemia-associated affections of NVU components were detected (e.g., Härtig et al., 2009; Michalski et al., 2010, 2018; Aleithe et al., 2019; Mages et al., 2019; Kestner et al., 2020). Furthermore, the blood-brain barrier (BBB) attracted interest as a highly dynamic part of the NVU (Yang et al., 2019). Away from the simplified perspective of a layer separating the blood from the brains’ parenchyma, the BBB rather represents a complex structure with endothelial cells and basement membranes as its main components (Dyrna et al., 2013). Especially in the setting of stroke, endothelial cells are believed to have a pivotal role in BBB integrity as the leakage of blood-sourced substances was found *via* a transendothelial route together with morphological features like an endothelial swelling (Krueger et al., 2013, 2015, 2019). Overall, abnormalities within the NVU with a consecutive impairment of the BBB integrity are known to increase the risk for secondary hemorrhage associated with recanalizing therapies, and thus critically impact the patients’ outcome (Yang and Rosenberg, 2011).

In addition to cellular alterations described in experimental studies, ischemia also affects the extracellular matrix (ECM; Härtig et al., 2017), which is known to have several properties concerning local hemostasis and neuronal function (Soleman et al., 2013). Among the subsumed non-cellular structures, the basement membrane is of special interest because of its close vicinity to the endothelial layer, leading to the assumption of an involvement in regulatory processes within the NVU and thus the BBB integrity (Reed et al., 2019; Xu et al., 2019). In addition to the proteins laminin, nidogen, and heparan sulfate proteoglycans, especially collagen isoforms with predominant collagen IV represent main components of the basement membranes (Thomsen et al., 2017; Gatseva et al., 2019). In this context, integrins were identified as transmembrane proteins with the two subunits *α* and *ß* linking the endothelial layer with the basement membrane (del Zoppo and Milner, 2006). Considering their special location, integrins can be seen as a connection between the vascular part of the NVU and the adjacent ECM, formerly described in terms of a vascular matrix adhesion complex (del Zoppo et al., 2006). Not surprisingly, integrins might have an important role in BBB stabilization also in the setting of stroke (Guell and Bix, 2014; Edwards and Bix, 2019). From the variety of ECM proteins, especially fibronectin, which provides a fibrillar matrix around cells (White et al., 2008; Singh et al., 2010), was perceived as highly reactive against ischemic stimuli (Edwards and Bix, 2019). Based on a modular composition and different domains, fibronectin interacts with other ECM proteins including other fibronectin molecules and also cell surface receptors (Singh et al., 2010; Mezzenga and Mitsi, 2019). Regarding the dynamic processes within the NVU and the associated ECM, fibronectin appears unique as its complex arrangement is mediated by integrins and other receptors located at the surface of for instance endothelial cells (Mao and Schwarzbauer, 2005; del Zoppo and Milner, 2006).

Overall, both the basement membrane and associated ECM proteins represent structures outside the cell-based NVU perspective that may sensitively react in the setting of ischemia. The few available data in the field indicated alterations of collagen IV and fibronectin after experimental stroke (Hamann et al., 1995), while simultaneous reactions of fibronectin and integrins were observed after focal (Huang et al., 2015) and global cerebral ischemia (Milner et al., 2008). Further, fibronectin serum levels of stroke patients were associated with hemorrhage as a typical complication following thrombolysis (Castellanos et al., 2004, 2007), suggesting an involvement in BBB dysregulations also in humans. However, additional data on these structures, not necessarily captured by the traditional NVU concept, are needed to generate a hypothesis regarding their functional relevance.

Therefore, the present study aims to explore ischemic consequences for the neurovascular matrix adhesion zone (NMZ) with a special focus on collagen IV as part of the basement membrane and adjacent fibronectin as part of the ECM. To consider potential spatio-temporal effects, quantitative analyses focused on diverse brain regions and different durations of ischemia after experimental stroke in mice. For translational issues, confirmatory analyses included experimental focal cerebral ischemia in rats, sheep, and human stroke tissue.

## Materials and Methods

### Study Design

Various combinations of fluorescence labeling were applied to explore ischemic consequences to the basement membrane and its regionally associated ECM including fibronectin and integrin α_5_ in conjunction with diverse components of the NVU, i.e., neurons, oligodendrocytes, micro‐ and astroglia as well as the vasculature. Details on the used brain tissues and models of focal cerebral ischemia are given below. Analyses were primarily focused on an ischemia duration of 4 h (*n* = 6) and 24 h (*n* = 8) in the mouse model, also used for quantification of collagen IV and fibronectin (*n* = 6 for 4 h, *n* = 5 for 24 h). Confirmative and thus qualitative analyses include an ischemia duration of 72 h in mice (*n* = 2), 24 h in rats (*n* = 3), and 2 weeks in sheep (*n* = 6) as well as 3 weeks in human stroke (*n* = 1). The reporting followed the ARRIVE guidelines for experimental research including animals (Kilkenny et al., 2010).

Animal experiments were carried out in accordance with the European Union Directive 2010/63/EU and the German guideline for care and use of laboratory animals. They had been approved by the Regierungspräsidium Leipzig as local authority (reference numbers: TVV 02/17 for mice and rats, and TVV 56/15 for sheep). For the reporting of histopathological findings in the human sections, prior consent was obtained as described by Kattah et al. (2017).

### Experimental Focal Cerebral Ischemia in Rodents

Adult male C57Bl/6J mice with a bodyweight of about 25 g, obtained by Charles River (Sulzfeld, Germany), underwent a filament-based model for permanent right-sided middle cerebral artery occlusion as originally described by Longa et al. (1989) with minor modifications (Hawkes et al., 2013). In brief, right-sided cervical arteries were carefully exposed while using an operation microscope. A standardized silicon-coated 6-0 monofilament (Doccol Corporation, Redlands, CA, United States) was inserted into the internal carotid artery and moved forward until bending was observed or resistance was felt (approximately 9 mm from carotid bifurcation). To induce permanent ischemia, the filament was left in place, and the skin was closed with a surgical suture. Mice were sacrificed at the end of an observation period of 4, 24, or 72 h after ischemia induction.

In adult male Wistar rats with a bodyweight of about 300 g, also provided by Charles River, a thromboembolic model was applied to obtain right-sided middle cerebral artery occlusion as originally described by Zhang et al. (1997) with minor modifications (Michalski et al., 2010). In brief, following the careful exposure of right-sided cervical arteries with an operation microscope, a polyethylene tube with tapered end was introduced into the external carotid artery and moved forward through the internal carotid artery (approximately 16 mm from carotid bifurcation). At this position, a rat blood-sourced clot was injected. Afterwards the catheter was removed, and the skin was closed with a surgical suture. Rats were sacrificed at the end of a 24-h observation period from ischemia induction.

In general, for surgical procedures, mice and rats were anesthetized using about 2–2.5% isoflurane (Isofluran Baxter, Baxter, Unterschleißheim, Germany; mixture 70% N_2_O/30% O_2_) with a commercial vaporizer (VIP 3000, Matrix, New York, United States) and received a complex pain medication. During surgical procedures, a thermostatically controlled warming pad with rectal probe (Fine Science Tools, Heidelberg, Germany) was used to prevent anesthesia-associated cooling, and the body temperature was kept stable at around 37°C. Sufficient induction of focal cerebral ischemia was ensured by the evaluation of individual neurobehavioral deficits, while animals had to present a score of at least 2 on the Menzies score (Menzies et al., 1992), ranging from 0 (no neuronal deficit) to 4 (spontaneous contralateral circling), which represents a pre-defined study inclusion criterion.

### Experimental Focal Cerebral Ischemia in Sheep

In male adult sheep (hornless Merino) with a bodyweight about 70 kg, provided by the Veterinary Faculty of Leipzig University (Lehr‐ und Versuchsgut Leipzig, Germany), middle cerebral artery occlusion was surgically induced as described by Nitzsche et al. (2016) and Boltze et al. (2008). Briefly, the left temporal bone was carefully exposed, and here, a trepanation with a 6 mm barrel burr at 10,000 rpm (Aesculap microspeed uni, Scil Animal Care Company GmbH, Viernheim, Germany) was performed. The dura mater was incised, and the middle cerebral artery was occluded at the distal M1 segment by electrosurgical coagulation using neurosurgical bipolar forceps (ME 411, KLS Martin, Tuttlingen, Germany). Finally, the dura mater was repositioned while muscles and the skin were sewed with surgical sutures. Sheep were sacrificed after an observation period of 2 weeks from ischemia induction.

For surgical procedure, sheep were anesthetized by an intravenous injection of ketamine (4 mg/kg body weight; Ketamin, Medistar, Holzwicke, Germany), xylazine (0.1 mg/kg body weight; Xylazin, Ceva Sante Animal GmbH, Düsseldorf, Germany), and diazepam (0.2 mg/kg body weight; Temmler Pharma GmbH, Marburg, Germany). Mechanical ventilation with 2% isoflurane and 40% oxygen (Primus, Dräger AG, Lübeck, Germany) allowed anesthesia during surgery. At the end of surgery, sheep were treated with the antibiotic enrofloxacin (5% Baytril, Bayer AG, Leverkusen, Germany) and the analgesic butorphanol (Alvegesic 1%; CP-pharm, Burgdorf, Germany). The presence of cerebral infarctions was confirmed by MRI.

### Human Stroke Tissue

Post-mortem brain tissue affected by an ischemic stroke was originally provided by Jorge C. Kattah (Department of Neurology, University of Illinois, College of Medicine, Peoria, IL, United States) in collaboration with Anja K.E. Horn (Institute of Anatomy and Cell Biology I, University of Munich, Germany). In detail, the autoptic brainstem sections contained ischemia-affected areas of the lateral medulla from a 61-year-old male who passed away 3 weeks after onset of stroke. Further data on the analyzed brain tissue and stroke characterization were summarized by Horn and co-workers in Kattah et al. (2017).

### Tissue Preparation

For fluorescence labeling, rodents were transcardially perfused after the respective observation period with saline and 4% phosphate-buffered paraformaldehyde (PFA). Brains were carefully removed from the skull and post-fixed in PFA overnight, followed by equilibration with 30% phosphate-buffered sucrose for a few days. Subsequently, forebrains from rodents were serially cut with a freezing microtome (Leica SM 2000R, Leica Biosystems, Wetzlar, Germany). Thereby, 10 series of 30 μm-thick sections each were collected.

Brains from sacrificed sheep were divided by producing about 10 mm thick slices. After their photo-documentation, each slice was immersion-fixed by 4% buffered formaldehyde for 14 days at 4°C and thereafter equilibrated in 30% phosphate-buffered sucrose. The immersion-fixed tissue blocs were consecutively cut at 40 μm thickness using a freezing microtome (Microm HM 430, Thermo Fisher Scientific, Waltham, MA, United States).

Until histochemical processing, sections from rodents and sheep were stored at 4°C in vials filled with 0.1M Tris-buffered saline (TBS), pH 7.4, containing sodium azide.

Human brain tissue was cut into 5 mm thick slices, fixed by formaldehyde and embedded in paraffin, further sectioned at 5 μm thickness and mounted onto microscope slides. For histochemistry, sections were deparaffinized in xylene and rehydrated in graded alcohols. Antigen retrieval was performed with 0.1M citrate buffer, pH 6, for 15 min in a water bath at 90°C.

### Histochemistry

Prior to all histological procedures, free-floating sections were extensively rinsed with TBS. All staining procedures started by blocking non-specific binding sites for subsequently applied immunoreagents with 5% normal donkey serum in TBS containing 0.3% Triton X-100 (NDS-TBS-T) for 1 h. Sections were then incubated for 20 h with mixtures of primary antibodies and biotinylated lectins – diluted in the blocking solution – as listed in Table 1. for all procedures, including goat-anti-collagen IV and rabbit-anti-fibronectin. Thereafter, sections were rinsed with TBS followed by incubation with mixtures of appropriate fluorochromated secondary immunoreagents [20 μg/ml TBS containing 2% bovine serum albumin (TBS-BSA); Dianova, Hamburg, Germany] according to Table 1.

**Table 1:** Triple fluorescence staining: marker combinations concomitantly applied with Cy3-stained rabbit-anti-fibronectin*.

First primary antibodies	First visualizing immunoreagents	Second primary antibodies or lectins	Second visualizing immunoreagents
sheep-anti-serum albumin (1:500; Serotec)	Cy2-donkey-anti-goat IgG	biotinylated LEA (20 μg/ml; Vector, Burlingame, CA, United States)	Cy5-streptavidin
goat-anti-collagen IV (1:100; Merck Millipore, Billerica, CA, United States)	Cy2-donkey-anti-goat IgG	biotinylated LEA (20 μg/ml; Vector)	Cy5-streptavidin
guinea pig-anti-NeuN (1:200; Synaptic Systems; Göttingen, Germany)	Cy2-donkey-anti-guinea pig IgG	biotinylated STL (20 μg/ml; Vector)	Cy5-streptavidin
guinea pig-anti-CNP (1:200; Synaptic Systems)	Cy2-donkey-anti-guinea pig IgG	biotinylated STL (20 μg/ml; Vector)	Cy5-streptavidin
guinea pig-anti-Iba (1:100; Synaptic Systems)	Cy2-donkey-anti-guinea pig IgG	biotinylated STL (20 μg/ml; Vector)	Cy5-streptavidin
guinea pig-anti-GFAP (1:200; Synaptic Systems)	Cy2-donkey-anti-guinea pig IgG	biotinylated STL (20 μg/ml; Vector)	Cy5-streptavidin
guinea pig-anti-GFAP (1:300; Synaptic Systems)	Cy2-donkey-anti-guinea pig IgG	biotinylated STL (20 μg/ml; Vector)	Cy5-streptavidin
goat-anti-collagen IV (1:100; Merck Millipore)	Cy2-donkey-anti‐ goat-IgG	guinea pig-anti-GFAP (1:200; Synaptic Systems)	Cy5-donkey-anti-guinea pig IgG
guinea pig-anti-AQP4 (1:100; Merck Millipore)	Cy2-donkey-anti-guinea pig IgG	goat-anti-collagen IV (1:100; Merck Millipore)	AlexaFluor647-donkey-anti-goat IgG

*Primary antibody: rabbit-anti-fibronectin (1:600, formerly AbD Serotec, Oxford, UK – now Bio-Rad, Munich, Germany); visualizing immunoreagent: Cy3-donkey-anti-rabbit IgG. All fluorescent immunoreagents were obtained from Dianova (Hamburg, Germany) and used at 20 μg/ml. LEA, biotinylated *Lycopersicon esculentum* agglutinin (= tomato lectin); STL, *Solanum tuberosum* agglutinin (= potato lectin); NeuN, neuronal nuclei; CNP, 2',3' cyclic nucleotide phosphodiesterase; Iba, ionized calcium binding adapter molecule-1; GFAP, glial fibrillary acidic protein; AQP4, aquaporin-4.

Additionally, sections from mice were blocked with NDS-TBS-T and processed for 20 h with an antibody cocktail consisting of sheep-anti-fibronectin (R&D Systems, Minneapolis, MN, United States; AF 1918, 1:200 in NDS-TBS-T), rabbit-anti-integrin *α*_5_ (Abcam, Cambridge, UK; ab221606, 1:100), and guinea pig-anti-glial fibrillary acidic protein (GFAP; Synaptic Systems, Göttingen, Germany; 1:200). After extensive rinses with TBS tissues were incubated for 1 h with a cocktail of Cy3-donkey-anti-goat IgG (highly cross-reacting with sheep IgG), Cy2-donkey-anti-rabbit IgG, and Cy5-donkey-anti-guinea pig IgG (all from Dianova; 20 μg/ml TBS-BSA).

In a first set of control experiments, the omission of primary antibodies or biotinylated lectins resulted in the expected absence of cellular labeling. Additional controls were the switch of fluorophores related to the concomitantly detected markers producing no alterations of their stainability. Moreover, immunolabeling of fibronectin with rabbit-anti-fibronectin from Serotec (4470-4339) that was used in the present study (and is now available from Bio-Rad, Munich, Germany) produced nearly the same staining patterns as sheep-anti-fibronectin (R&D) and rabbit-anti-fibronectin from Abcam (ab2413).

Finally, all free-floating sections were extensively rinsed with TBS and briefly with distilled water, mounted onto glass slides, air-dried, and coverslipped with Entellan in toluene (Merck, Darmstadt, Germany). In parallel, stained human paraffin sections underwent a Sudan black B post-treatment for 10 min to quench their autofluorescence. These sections were coverslipped with glycerol gelatin (Sigma, Taufkirchen, Germany).

### Microscopy, Image Processing, and Quantification of Fluorescence Signals

For the subsequent imaging process of selected brain regions with different magnifications, the microscope Biorevo BZ-9000 (Keyence, Neu-Isenburg, Germany) was used. Panels of micrographs were generated with Microsoft PowerPoint (Office 365, Version 2018; Microsoft Corp., Redmond, WA, United States). If necessary, brightness and contrast of micrographs were slightly adjusted without the deletion or creation of signals.

Quantitative analyses of the immunofluorescence signals from collagen IV and fibronectin were based on five brain sections from each mouse, while these sections were selected concerning the following criteria: first, an unequivocal ischemic affection as indicated by a regional change of the fluorescence signal typically located in the right-sided neocortex and subcortical areas as known from own earlier studies (e.g., Mages et al., 2018), and second, the absence of tissue damage that prevents immunofluorescence-based analyses in large parts of the ischemia-affected hemisphere. This selection process allowed the analysis of six mice with an ischemia duration of 4 h and five mice with an ischemia duration of 24 h. As prerequisite for the following quantification, in each of the selected brain sections, seven regions of interests (ROIs) were arranged on the ischemia-affected hemisphere including six neocortical ROIs covering the ischemic border zone with most evident changes of the collagen IV immunosignal between ROIs 3 and 4, and a further ROI (number 7) in the subcortex, i.e., the striatum. For control measurements, seven additional ROIs were mirrored on the contralateral, non-affected hemisphere. This method collectively results in 14 ROIs per brain section, while each ROI was captured with a single image using a 40x objective on the Biorevo microscope. The exposure time was routinely adjusted at the level of each section to avoid overexposure, and at the same time, to allow inter-hemispheric comparison. This technique resulted in usually 70 images from each animal, but due to porous infarct material in one mouse, only 60 images were available. Overall, 760 images from 11 mice were included in quantitative analyses.

Immunofluorescence signals of fibronectin were captured by mean values in the obtained images from each ROI using ImageJ (National Institutes of Health, Bethesda, MD, United States). As measuring method for collagen IV, the maximum immunosignal intensity was used to consider the strongly vessel-associated signal that was accompanied by a visually darker background on the affected hemisphere compared to the contralateral side. Finally, rounded mean values of the obtained fluorescence signals of collagen IV and fibronectin were calculated for every ROI of the five sections at the level of each animal. Further calculations included both an inter-hemispheric comparison with reference to ROIs along the ischemic border zone in the neocortex and the striatum in the overall sample, as well as differences between the ischemia‐ and non-affected hemispheres used for analyses concerning potential time-dependent effects in respective subgroups.

### Statistical Analyses

The SPSS software package version 24 (IBM SPSS Statistics for Windows, IBM Corp., Armonk, NY, United States) was used for descriptive analyses and testing concerning statistical significance between groups. Because of the relatively small sample size, non-parametric tests were applied, i.e., the Wilcoxon test and the Mann-Whitney *U* test. To consider multiple testing, the Bonferroni-Holm correction was added to these tests. Further, Pearson correlation coefficients (r) were used to explore statistical relationships between parameters, added by a calculation of the explained variance (*r*^2^). Generally, a value of *p* < 0.05 (if applicable after correction for multiple testing) was considered as statistically significant.

## Results

### Ischemic Consequences to the Vascular Integrity and Extracellular Matrix

**Figure 1:**
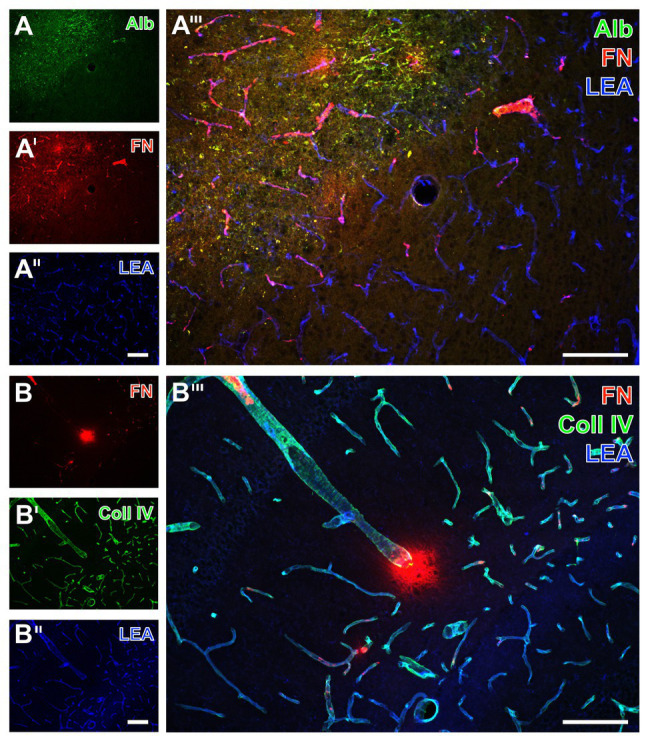
Fluorescence-based visualization of impaired blood-brain barrier integrity with vascular elements as well as collagen IV and fibronectin in mice subjected to 24 h of focal cerebral ischemia. Extravasation of (endogenous) serum albumin (Alb, **A**) into brains’ parenchyma is visible in conjunction with an increased immunosignal of fibronectin (FN, **A'**) as a component of the extracellular matrix (ECM). FN also appears in close vicinity to collagen IV (Coll IV, **B'**) as part of the basement membrane 24 h after ischemia onset. FN is thereby visible in the striatum **(A)** and the hippocampus **(B)**. In the area of ischemia, immunosignals for Alb **(A)** and fibronectin **(A',B)** are associated with endothelia, as detected by the lectin-staining with LEA **(A'',B'')**, and are visible within the adjoining neuropil in a carpet-like manner. Ischemia-affected tissue selectively exhibits a strong immunosignal for Coll IV **(B')**. The overlay of staining patterns clearly reveals the border of ischemic affection **(A''')** and different forms of vessel-associated focal condensation of FN. Scale bars: in **A'''** and **B'''** = 100 μm, in **A''** (also valid for **A** and **A'**) and **B''** (also valid for **B** and **B'**) = 100 μm.

Twenty-four hours of focal cerebral ischemia in mice resulted in a critical affection of the BBB integrity as visualized by an extravasation of serum albumin into the parenchyma in ischemic areas (left upper part in Figure 1A), whereas no albumin extravasation was visible in non-affected areas. Concerning the vasculature, lectin-based staining with the tomato lectin (*Lycopersicon esculentum* agglutinin, LEA) showed a homogeneous pattern of vessels in ischemia‐ and non-affected areas (Figures 1A'',B'). While addressing collagen IV as a major component of basement membranes, an increased and strongly vessel-associated immunosignal became evident in areas affected by ischemia (Figures 1B',B'''). Concerning fibronectin as part of the ECM, an increased immunosignal was routinely observed in ischemia-affected areas (Figures 1A',A''') with both a strongly vessel-associated pattern and a diffuse appearance in close vicinity to the vasculature not necessarily overlapping with cellular structures (Figures 1A''',B'''). These fluorescence-based staining patterns of collagen IV and fibronectin were consistently found in ischemia-affected subcortical, i.e., the striatum, and hippocampal areas (Figures 1A,B), as well as the ipsilateral neocortex (not shown).

**Figure 2:**
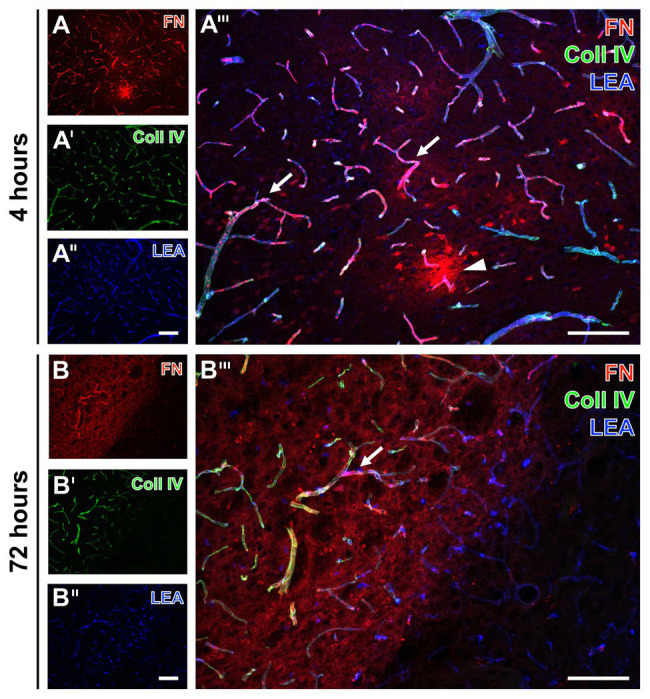
Fluorescence-based detection of collagen IV and fibronectin combined with vessel staining in mice subjected to 4 and 72 h of experimental focal cerebral ischemia. Increased immunosignals of fibronectin (FN) and collagen IV (Coll IV) are visible at both 4 **(A)** and 72 h **(B)** of ischemia in affected areas. At both time points, FN consistently appears associated with the vasculature (arrows in **A'''** and **B'''**) and in a diffuse manner with focal accumulations (arrow head in **A'''**) in close vicinity to vessels. Concomitantly, increased Coll IV immunosignals precisely demarcate the area of ischemic affection **(B')**, which becomes even clearer when merging the staining patterns of FN and Coll IV **(B''')**. Thereby, overlapping signals of FN and Coll IV becomes visible by the purple color **(A''',B''')**. Lectin-based counterstaining using LEA visualizes the vasculature also in ischemic areas **(A'',B'')**. Scale bars: in **A'''** and **B'''** = 100 μm, in **A''** (also valid for **A** and **A'**) and **B''** (also valid for **B** and **B'**) = 100 μm.

To explore potential time-dependent alterations, analyses were extended to ischemic tissues from mice subjected to 4 and 72 h of focal cerebral ischemia. In accordance to the findings at 24 h, increased immunosignals of collagen IV and fibronectin were found very early and also 3 days after ischemia onset (Figure 2). Thereby, the ischemia-affected striatum displayed an increased immunosignal of fibronectin that was associated with the vasculature as indicated by an overlapping lectin signal (arrows in Figures 2A''',B'''). The immunosignal of fibronectin also appeared in a diffuse pattern with some focal accumulations in close vicinity to the vasculature (arrowhead in Figure 2A'''). In a more general perspective, the increased fibronectin signal robustly demarcated the area of ischemia (left upper part in Figures 2B,B'''). As already demonstrated at 24 h after ischemia onset, lectin-based staining robustly visualized vessels also in areas not affected by ischemia (right bottom in Figures 2B'',B''').

### Alterations of Collagen IV and Fibronectin Depending on Brain Region and Duration of Ischemia

**Figure 3:**
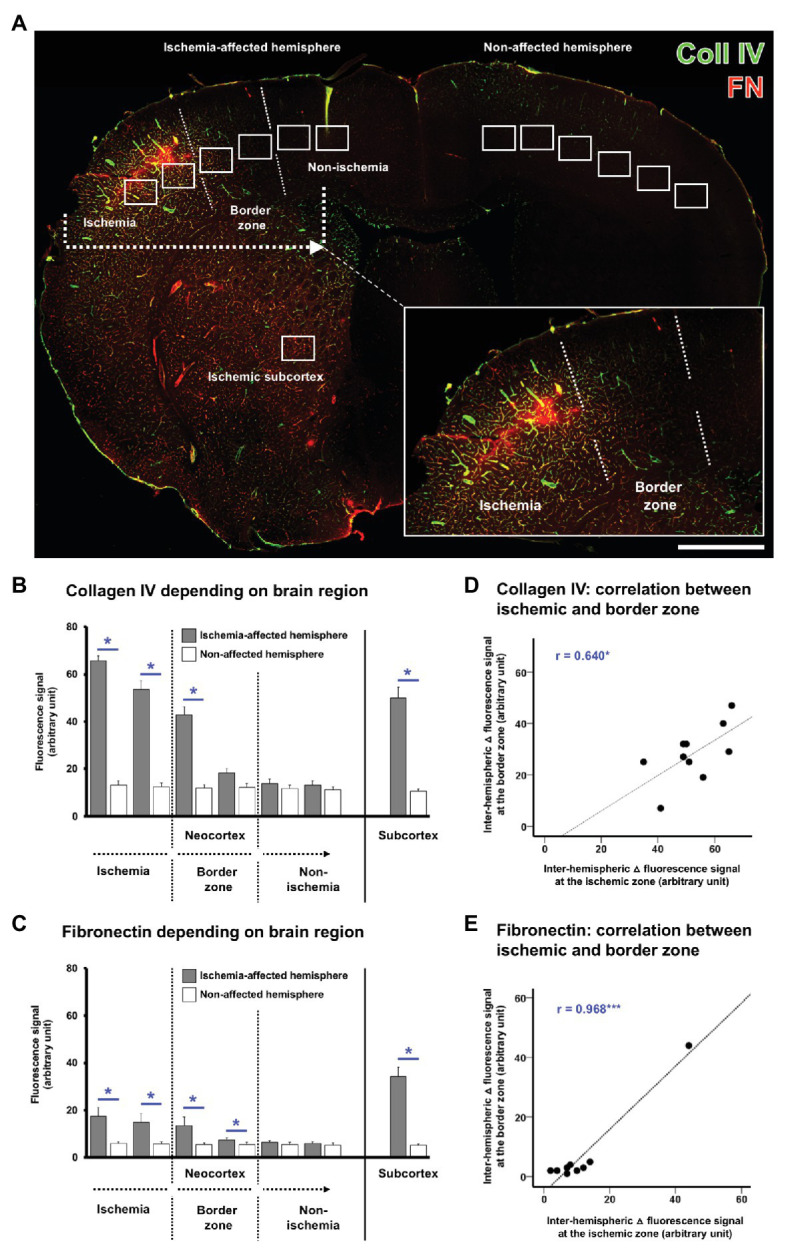
Quantification of collagen IV and fibronectin immunosignals in the overall sample of mice subjected to 4 or 24 h of experimental focal cerebral ischemia. Region-related quantification of both immunosignals based on seven regions of interest (ROIs) located on the ischemia-affected hemisphere, while seven ROIs mirrored to the contralateral, i.e., non-affected hemisphere served as control **(A)**. The ischemia-affected neocortex is captured by six ROIs with the ischemic border zone in the middle part, and the subcortical region is captured by a single ROI **(A)**. Immunosignals of collagen IV **(B)** and fibronectin **(C)** are significantly increased in the neocortical area of maximum ischemic affection with a gradual decline toward the ischemic border zone and the non-affected region. A significantly increased immunosignal is also seen in the subcortical region **(B,C)**. Correlation plots including the area of maximum ischemic affection and the ischemic border zone indicate a strong positive correlation between these regions for the inter-hemispheric differences of collagen IV **(D)** and fibronectin **(E)** indicating commutated changes in both regions at the same time. Scale bar: in **A** = 1 mm. Bars represent means and added lines represent the standard error of means. *r*: Pearson correlation coefficient. Levels of statistical significance: ^*^p < 0.05, ^***^p < 0.001.

With the intention to verify the observed alterations in the immunosignals of collagen IV and fibronectin, quantitative analyses were focused on the ischemic border zone in the neocortex and the ischemia-affected subcortex. For this purpose, on the ischemia-affected hemisphere, six ROIs were used to capture the ischemic border zone in the neocortex, and a further ROI was applied to capture the ischemic striatum, whereby each of the ROIs was mirrored to the contralateral, non-affected hemisphere for control issues (Figure 3A).

In the overall sample used for quantification of mice subjected to 4 and 24 h of focal cerebral ischemia, a gradual decrease was observed for the collagen IV immunoreactivity with a maximum signal in the area of ischemia and the most considerable step toward an immunosignal that is comparable with the non-affected hemisphere in the ischemic border zone (Figure 3B). Inter-hemispheric comparison led to significant differences in the first three ROIs starting in the ischemic area with value of *p* corrected for multiple testing between 0.021 and 0.025. When compared to the non-affected hemisphere, a similar immunosignal of collagen IV was found for the most medial ROIs, assumed to be non-altered by ischemia. Along the ischemia-affected neocortex, the stepwise decline of collagen IV immunoreactivity was found as significant between the first (lateral, and thus most ischemia-affected) and the second ROI (*p* = 0.020), the second and the third ROI (*p* = 0.020), as well as the third and the fourth ROI (*p* = 0.015). Subcortically, a significantly increased collagen IV immunosignal was identified in the striatum as compared with the contralateral site (*p* = 0.021).

A quite similar pattern was observed for fibronectin with a gradual decrease of the immunoreactivity, starting with its maximum in the area of ischemia, and followed by a considerable step in the ischemic border zone, ultimately resulting in signals with good accordance to the non-affected hemisphere (Figure 3C). Inter-hemispheric comparison revealed significant differences in the first four ROIs completely covering the area of ischemia and the ischemic border zone with value of *p* ranging from 0.021 to 0.030. Along the ischemia-affected neocortex, the immunosignal of fibronectin declined in a stepwise manner from the first (most ischemia-affected) to the second ROI (*p* = 0.028) as well as from the third to the fourth ROI (*p* = 0.015). In the subcortical region, a significantly increased fibronectin immunosignal was found as compared to the non-affected site (*p* = 0.021).

Subsequent analyses addressed the correlation between different regions within the ischemia-affected neocortex to explore the statistical relationship, and thus simultaneously occurring changes beyond a single region. For both collagen IV (Figure 3D) and fibronectin (Figure 3E), a significant correlation was observed between the most severely affected ROI and the ischemic border zone, indicating commutated alterations with an explained variance of 40.9% for collagen IV and remarkable 93.7% for fibronectin.

**Figure 4:**
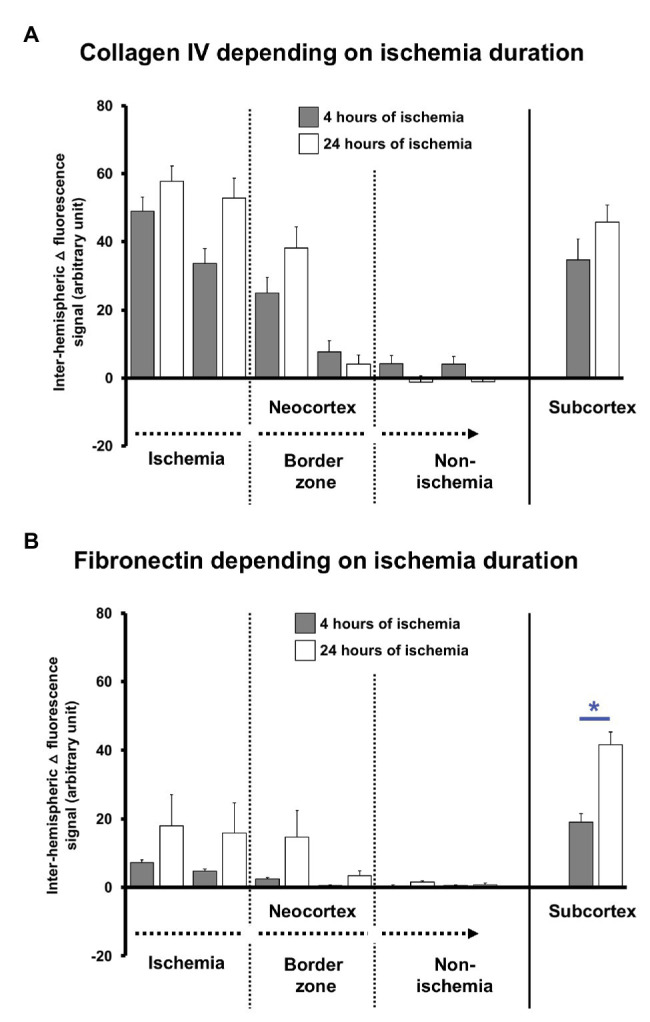
Quantification of collagen IV and fibronectin immunosignals in mice depending on the duration (4 vs. 24 h) of experimental focal cerebral ischemia. Inter-hemispheric differences covering the immunosignals of collagen IV **(A)** and fibronectin **(B)** depending on the duration of ischemia confirms the gradual decrease of the signals along the ischemia-affected neocortex as evaluated in the overall sample. Statistically significant differences are lacking between the 4 and 24 h of ischemia in the neocortex, although a trend is visible for a pronounced signal at the later time point. In the subcortex, the immunosignal of fibronectin increased at 24 h as compared with the earlier time point. Bars represent means and added lines represent the standard error of means. Level of statistical significance: ^*^p < 0.05.

To explore potential time-dependent effects, altered immunosignals of collagen IV and fibronectin along the ischemia-affected neocortex and the subcortex were separately analyzed for 4 and 24 h of ischemia. Thereby, the inter-hemispheric differences (△) of the collagen IV immunosignals confirmed the gradual decline from the most-affected toward the non-affected neocortex without significant differences between the two time points (Figure 4A). However, in most affected neocortical regions and the subcortex, the collagen IV immunoreactivity appeared at least numerically increased 24 h after ischemia when compared to the earlier time point (value of *p* ranged between 0.378 and 0.568). A comparable pattern was observed for the immunosignal of fibronectin that shows a gradual decline along the ischemia-affected neocortex toward non-affected areas without a statistically significant difference between the two time points (Figure 4B), while a numeric increase became noticeable in areas of ischemic affection (value of *p* ranged between 0.120 and 0.724). Remarkably, in the ischemia-affected subcortex, the fibronectin immunoreactivity was significantly increased after 24 h of ischemia when compared to 4 h (*p* = 0.042).

### Spatial Relationships Between Fibronectin and the Neurovascular Unit

**Figure 5:**
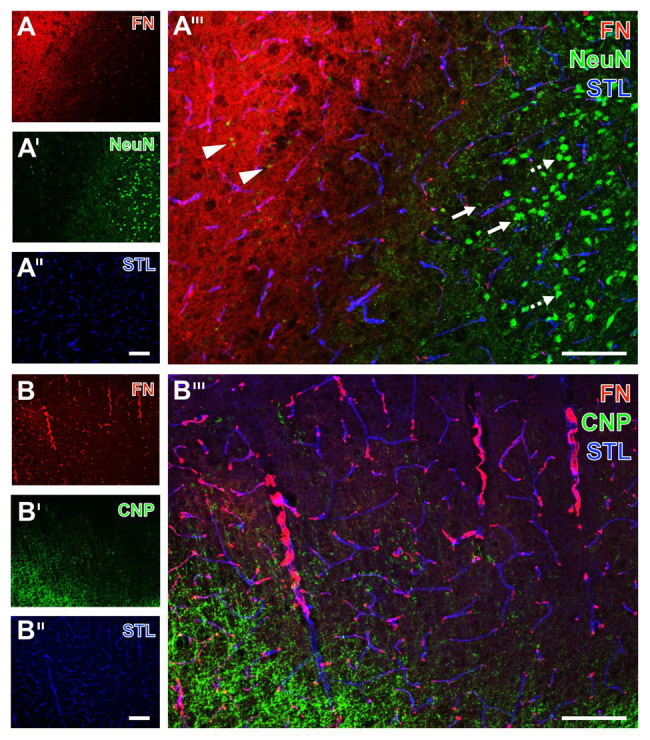
Spatial relationships between fibronectin, the vasculature, neurons, and oligodendroglia in mice subjected to 24 h of experimental focal cerebral ischemia. Staining of fibronectin (FN, **A,B**) and striatal endothelia visualized by a lectin-based technique using *Solanum tuberosum* lectin (STL, **A'',B''**) combined with the detection of neurons (NeuN, **A'**) show a regular configuration of NeuN in the non-affected area (arrows with dashed lines in **A'''**). Significant neuronal degeneration is visible toward the ischemic region with a co-occurring increase of the FN signal, starting in the ischemic border zone with shrunken NeuN (arrows in **A'''**) and passing over to the area of maximum ischemic affection with only fragments of NeuN (arrowheads in **A'''**). The concomitant visualization of oligodendroglia in the ischemia-affected neocortex reveals an increased immunosignal of the applied marker CNP **(B')**, not overlapping with FN **(B''')**. Scale bars: in **A'''** and **B'''** = 100 μm, in **A''** (also valid for **A** and **A'**) and **B''** (also valid for **B** and **B'**) = 100 μm.

**Figure 6:**
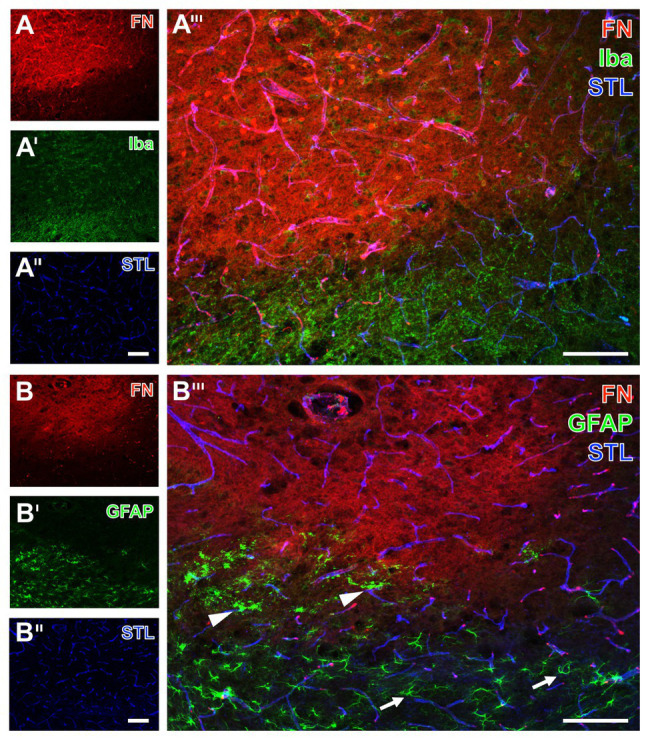
Spatial relationships between fibronectin, the vasculature, microglia, and astrocytes in mice subjected to 24 h of experimental focal cerebral ischemia. Staining of fibronectin (FN, **A,B**) together with the lectin-based visualization of the endothelium by STL **(A'',B'')** and Iba **(A')** as marker for microglia indicate a mainly morphological alteration of the latter one in the ischemia-affected striatum. Toward the area of ischemia an increase of the FN signal is visible **(A)** in addition to nearly unaltered detection of vessels **(A'')**. GFAP immunolabeling displays a loss of astrocytes in the striatum as the region of maximum ischemic affection **(B')** with a simultaneous increase of the FN signal **(B)**. In the ischemic border zone, astrocytes are morphologically altered (arrow heads in **B'''**) when compared to their regular appearance (arrows in **B'''**). Scale bars: in **A'''** and **B'''** = 100 μm, in **A''** (also valid for **A** and **A'**) and **B''** (also valid for **B** and **B'**) = 100 μm.

To explore spatial associations to classical components of the NVU, qualitative analyses based on multiple fluorescence labeling on tissues originating from mice subjected to 24 h of focal cerebral ischemia were added (Figures 5, 6).

Starting with neurons, the immunosignal of neuronal nuclei (NeuN) clearly diminished in the area of ischemic affection (left upper part in Figure 5A'), while that of fibronectin became visible in a diffuse manner with some focal accumulations and vascular associations. In contrast to the area not affected by ischemia with strong and regular labeling of NeuN (arrows with dashed line in Figure 5A'''), signs of neuronal degradation (arrows) and ultimately shrunken NeuN became visible toward the ischemic area (arrowheads in Figure 5A'''). Concomitant staining with *Solanum tuberosum* lectin (STL) enabled (like the tomato lectin LEA) a robust, nearly unaltered visualization of the vasculature (Figures 5A'',A''').

To address oligodendrocytes as part of the glia within the NVU, multiple fluorescence labeling was used to detect the 2',3' cyclic nucleotide phosphodiesterase (CNP) in conjunction with fibronectin and STL. While fibronectin was again found to exhibit a vessel-associated appearance as indicated by the overlapping lectin signal (purple color in Figure 5B'''), the ischemia-related increase of the CNP immunoreactivity was observed somewhat distant from fibronectin without evidence for overlapping structures.

As further parts of the glia network, microglia and astrocytes were visualized using the ionized calcium binding adapter molecule-1 (Iba) and GFAP concomitantly with fibronectin immunolabeling. Thereby, microglia were found to be altered with predominantly morphological features in terms of a rounded appearance of GFAP-positive structures together with an overall weakened signal in the ischemia-affected area (upper left part of Figure 6A'). As expected, Iba-positive microglia were found unaltered in the non-affected regions (bottom right of Figure 6A') as indicated by their ramified appearance. Concerning astroglia, more drastic changes became visible by a nearly disappearing GFAP immunoreactivity in the area of maximum ischemic affection (upper part of Figure 6B'), while morphological alterations were evident in the ischemic border zone (arrowheads in Figure 6B'''), and the typically branched astroglia was visible in the non-affected region (arrows in Figure 6B'''). Remarkably, an overlapping immunoreactivity for fibronectin as well as microglia and astroglia was not evident (Figures 6A''',B''').

### Ischemic Consequences to Collagen IV and Fibronectin in Other Animal Species

**Figure 7:**
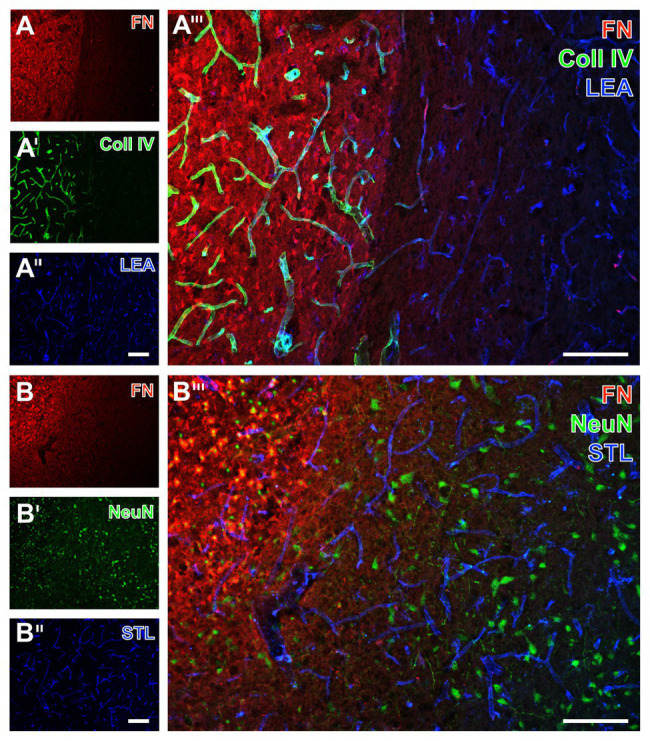
Immunofluorescence-based visualization of fibronectin together with vasculature elements and neurons in rats subjected to 24 h of experimental focal cerebral ischemia. Detection of fibronectin (FN, **A**) in conjunction with collagen IV (Coll IV, **A'**) and neurons using NeuN **(B')** as well as the vasculature as visualized by a lectin-based technique using LEA **(A'')** and STL **(B'')** in the ischemia-affected striatum confirms concomitantly increased immunosignals of FN and Coll IV **(A''')** also in the rat, and neuronal degeneration due to ischemia by an altered NeuN staining in the affected area **(B''')**. Both LEA and STL robustly visualize vessels also in ischemic areas, although STL leads to a somewhat thinner appearance of vessels in ischemic regions **(B''')**. Scale bars: in **A'''** and **B'''** = 100 μm, in **A''** (also valid for **A** and **A'**) and **B''** (also valid for **B** and **B'**) = 100 μm.

**Figure 8:**
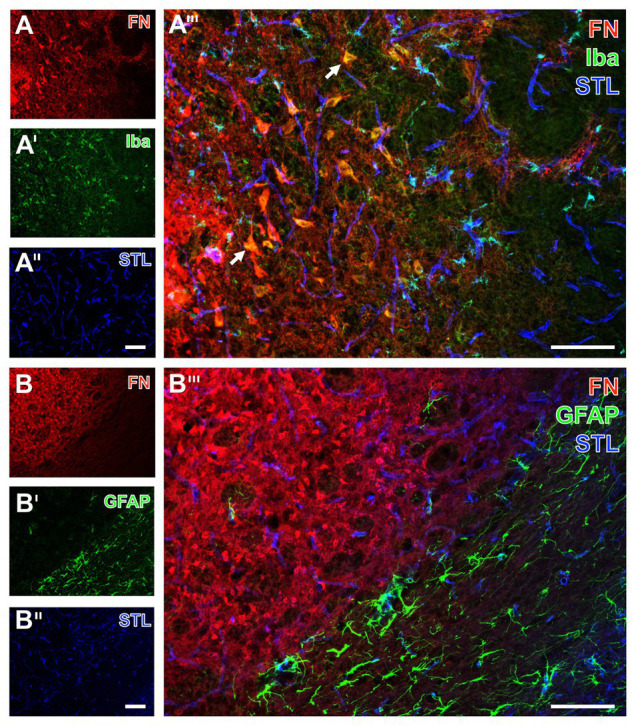
Immunofluorescence-based visualization of microglia and astrocytes combined with fibronectin and vascular elements in rats subjected to 24 h of experimental focal cerebral ischemia. Staining of fibronectin (FN, **A,B**) and lectin-based visualization of vessels using STL **(A'',B'')** together with microglial Iba **(A')** and astroglial GFAP **(B')** confirms for the rat striatum the predominantly morphological alterations of microglia and the nearly disappearing astrocytes in ischemic regions also seen in mice. FN robustly demarcates the area of ischemia **(A,B)**, while the merge with the staining pattern originating from microglia displays an only occasional overlap with the Iba signal (arrow in **A'''**). In contrast, STL appears evenly distributed in differently affected tissue as visualized by merging the staining patterns **(A''',B''')**. Scale bars: in **A'''** and **B'''** = 100 μm, in **A''** (also valid for **A** and **A'**) and **B''** (also valid for **B** and **B'**) = 100 μm.

**Figure 9:**
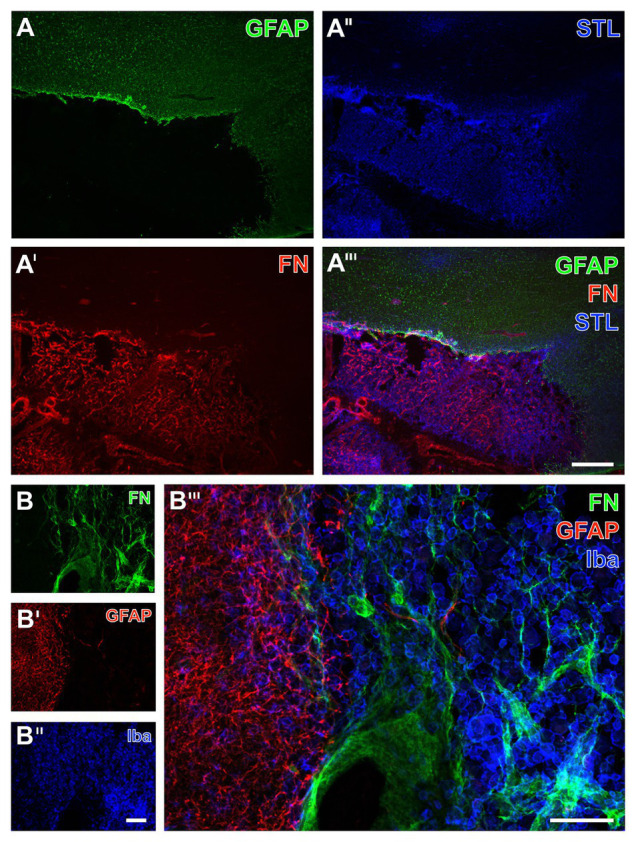
Immunofluorescence-based detection of fibronectin, micro‐ and astroglia as well as the vasculature in sheep subjected to 14 days of experimental focal cerebral ischemia. Numerous astrocytes as visualized by the GFAP **(A)** with a condensation at the ischemic border zone probably indicate the expected glia scar **(A)**, which is strictly separated from the immunosignal of fibronectin (FN, **A'**) that appears in the ischemic area. As revealed by potato lectin (STL, **A''**), this area also contains several cells presumably representing macrophages in addition to endothelial structures. The overlay verifies the complementary staining patterns of FN and the GFAP-positive astroglia **(A''')**. At higher magnification, the FN immunoreactivity remains not restricted to vessels but is also visible in the surrounding tissue **(B)**; however, nearly disappearing in the area covered by GFAP-positive astroglia **(B')**, which becomes clearly visible in the overlay of staining patterns **(B''')**. Concomitant labeling with antibodies directed against Iba **(B'')** visualizes microglia and mainly infiltrated macrophages in the area of ischemia affection that is immunopositive for FN **(B,B''')**. Scale bars: in **A'''** (also valid for **A**, **A'**, and **A''**) = 500 μm, in **B'''** = 100 μm, in **B''** (also valid for **B** and **B'**) = 100 μm.

With the intention to verify the identified regional arrangement of collagen IV and fibronectin as well as elements of the NVU in a translational perspective, further qualitative analyses included multiple immunofluorescence labeling on tissues originating from rats subjected to 24 h of an embolic-sourced focal cerebral ischemia (Figures 7, 8) and sheep subjected to 2 weeks of surgically induced focal cerebral ischemia (Figure 9). In general, the obtained data largely resembled the findings observed in the filament-based mouse model of focal cerebral ischemia as described above. They are exemplified for the striatum, while the similar data of the neocortex are not shown.

In the ischemia-affected striatum of rats, a strong collagen IV immunoreactivity was robustly found to demarcate the ischemic area that is also characterized by an enhanced fibronectin signal, appearing in a diffuse manner and some focal accumulations (left part in Figures 7A,A'). In contrast, in the area that is not affected by ischemia, the vasculature was visualized by LEA (Figure 7A''), whereas collagen IV immunoreactivity was widely lacking and fibronectin only displayed a considerably weakened signal toward the ischemic border zone (Figure 7A'''). Labeling of NeuN revealed diminished immunosignals and morphologically altered nuclei within the area of ischemic affection that was characterized by an increased fibronectin immunoreactivity (upper left part in Figures 7B,B').

For the visualization of microglia and astroglia in the rat, the detection of Iba and GFAP was combined with fibronectin immunolabeling. In line with the data originating from mice, Iba immunoreactivity indicated morphologically altered microglia in the area of ischemic affection, whereas fibronectin showed increased immunosignals with focal accumulations (Figures 8A,A'). Additionally, the ischemia-affected area also displayed a few overlapping signals of fibronectin and Iba (arrows in Figure 8A'''). Concerning astroglia, a nearly abolished immunosignal of GFAP was observed in the ischemic area along with an increased signal for fibronectin (Figures 8B,B'). Thus, overlapping signals of GFAP and fibronectin were not found (Figure 8B''').

To verify the observed alterations of fibronectin in a further species and to gain insights into a later time point following ischemia onset, multiple immunofluorescence labeling was applied to the ischemia-affected neocortex of sheep. At lower magnification, a strong GFAP immunoreactivity was observed at the ischemic border zone probably representing a part of the glia-based scar adjacent to the ischemic area devoid of GFAP (bottom of Figure 9A). Remarkably, concomitant staining of fibronectin revealed a strong immunosignal restricted to the area of ischemia (Figure 9A'), whereas the STL-based staining of the vasculature – and in this case additional cells – yielded a pronounced signal toward the ischemic region and did not completely disappear in non-affected areas (Figure 9A''). Triple immunofluorescence labeling further showed the presence of morphologically changed ameboid Iba-positive microglia as well as round cells assumed to represent immigrated macrophages in the ischemia-affected area. This tissue was devoid of GFAP-positive astroglia but exhibited strong fibronectin immunoreactivity with both a diffuse appearance and some focal accumulations (Figure 9B'''). Overall, in good accordance to the above described findings in rodents, fibronectin and the GFAP-immunoreactive astroglia were found to provide a complementary staining pattern in sheep too (Figures 9A''',B''').

### Ischemia-Related Alterations of Collagen IV and Fibronectin in Human Stroke

**Figure 10:**
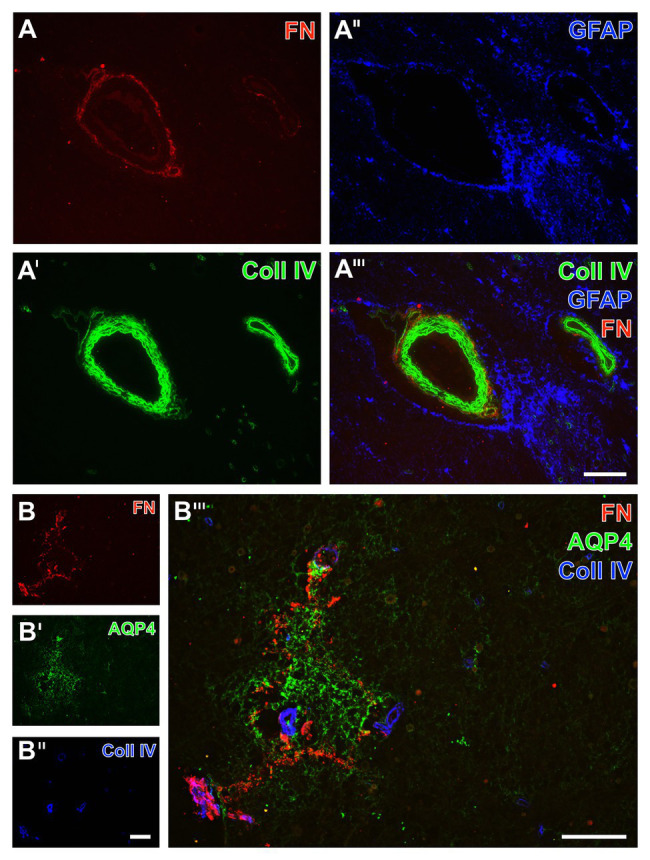
Triple immunofluorescence labeling of fibronectin, collagen IV, and astrocytes in human autoptic tissue affected by a stroke with a post-ischemic period of 3 weeks. Staining of fibronectin (FN, **A,B**) and collagen IV (Coll IV, **A',B''**) combined with astroglial components, i.e., GFAP **(A'')** located in cell bodies and AQP4 typically located in astroglial endfeet (AQP4, **B'**), reveals a shell-like pattern of FN and Coll IV in the ischemia-affected brainstem, while the FN signal is encircling Coll IV as obvious at perpendicularly cut vessels **(A''')**. In close vicinity to the vessels, diffusely labeled GFAP-containing astroglial structures are visible **(A'',A''')**, not overlapping with FN or Coll IV. The AQP4 immunoreactivity appears in an inhomogeneous manner not following cellular compartments as a sign of significantly degenerated astroglial endfeet in close regional association to the ischemia-related increase of FN signal **(B''')**. Scale bars: in **A'''** (also valid for **A**, **A'**, and **A''**) = 100 μm, in **B'''** = 100 μm, in **B''** (also valid for **B** and **B''**) = 100 μm.

Autoptic human stroke tissue was used to consider translational aspects of the observed ischemia-related alterations of fibronectin and collagen IV in rodents and sheep (Figure 10). In the ischemia-affected area, fibronectin was found strongly associated to vessels and their basement membranes as collagen IV immunoreactivity was regularly found to be encircled by the fibronectin signal, which became exemplarily visible in perpendicularly cut vessels (Figures 10A,A'). Astroglia, visualized by GFAP, were arranged farthest from the basement membrane (Figure 10A''').

As an approach to explore regional associations of astrocytes as well as fibronectin and collagen IV in more detail, subsequent triple immunofluorescence labeling included aquaporin 4 as an established marker for astrocyte endfeet (Figure 10B). In the area of ischemia, the immunosignal of fibronectin regularly appeared in a diffuse manner with some focal accumulations (Figure 10B). With reference to the immunosignal of fibronectin, aquaporin-4 (AQP4)-positive astrocytic structures became visible in close regional association in the ischemic area (Figure 10B'''), whereas their signal appeared in a disarranged pattern indicating morphologically altered endfeet in terms of cellular degenerations.

### Spatial Arrangement of Fibronectin and Integrin α_5_

**Figure 11:**
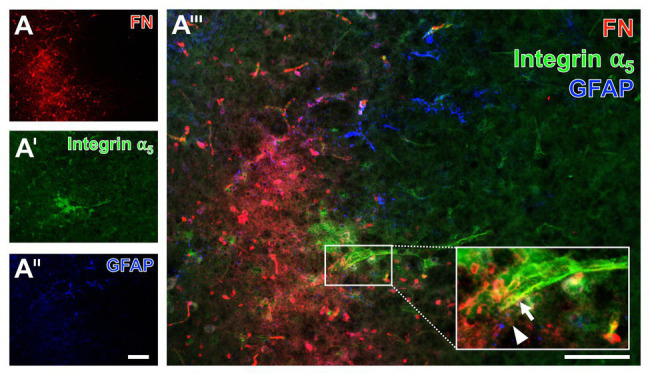
Immunofluorescence labeling of fibronectin and associated integrin *α*_5_ combined with the detection of astroglia in a mouse suffering from 72 h of experimental focal cerebral ischemia. Triple immunofluorescence labeling of fibronectin (FN, **A**), integrin *α*_5_ **(A')**, and astroglia as identified by GFAP **(A'')** confirms the increased immunosignal of FN in the ischemia-affected striatum in an expanded time window of 3 days after ischemia onset. The immunosignal of GFAP-positive astrocytes is clearly diminished in the area of ischemia **(A'')**. FN and integrin *α*_5_ appear vessel-associated, which becomes even clearer at a higher magnification (integrin *α*_5_: arrow in **A'''**, FN: arrowhead in **A'''**). Scale bars: in **A'''** = 100 μm, in **A''** (also valid for **A** and **A'**) = 100 μm.

Given the fact that integrins are seen as connecting proteins between the endothelial layer and the ECM with its special component fibronectin, an additional approach focused on their spatial arrangement under ischemic conditions. For this purpose, triple immunofluorescence labeling, also including astroglial GFAP that has shown to demarcate the affected area, was applied to the brain tissue of a mouse subjected to filament-based focal cerebral ischemia with an observation period of 3 days (Figure 11). Thereby, an increased fibronectin immunoreactivity consistently appeared in the above described diffuse manner with some focal accumulations in the area of ischemic affection, whereas the GFAP-positive astroglia was mostly lacking (left bottom part of Figure 11A). In the area of ischemic affection, the integrin α_5_ immunoreactivity appeared in a weak and diffuse pattern, while a regional accumulation was seen associated with the vasculature (Figure 11A') that became even more visible at a higher magnification (arrow in Figure 11A'''). This magnification also confirmed the vessel-associated pattern of the fibronectin immunosignal (arrowhead in Figure 11A''').

## Discussion

This study addressed ischemic consequences beyond the traditional cellular-based perspective with a special focus on the NMZ, representing the composite of NVU elements, i.e., the vasculature with its endothelium and the adjoined basement membrane, and the ECM (del Zoppo and Milner, 2006; Reed et al., 2019; Xu et al., 2019). Due to limited knowledge regarding the NMZ, neuroprotective approaches have not yet considered this structure, although a strong relationship to the integrity of the BBB with far reaching consequences is deducible already from its physical composition (del Zoppo et al., 2006).

Using multiple immunofluorescence approaches to tissues affected by different durations of focal cerebral ischemia, a spatio-temporal characterization of collagen IV as the major part of the basement membrane (Thomsen et al., 2017; Gatseva et al., 2019), and fibronectin as a major component of the ECM (Singh et al., 2010; Mezzenga and Mitsi, 2019) was performed. To consider the phenomenon of a translational roadblock in the field of stroke research that was at least partially related to artificial models (Young et al., 2007; Endres et al., 2008), efforts have been made to use different animal models with individual strengths and weaknesses (Durukan and Tatlisumak, 2007) as well as different species for this study. Having in mind that some shortcomings naturally remain, i.e., concerning the comparability of animal and human stroke data (Fisher et al., 2009; Sommer, 2017), also human tissue was included in histochemical analyses.

### Histomorphological Characterization of Collagen IV and Fibronectin After Ischemia

In the present study, qualitative analyses including different ischemia models and animal species consistently demonstrated visually increased immunosignals of collagen IV and fibronectin in areas affected by focal cerebral ischemia. As expected, collagen IV was associated with the vasculature, whereas fibronectin provided a diffuse staining pattern with focal accumulations in close vicinity to vessels. This finding on collagen IV largely confirms earlier reports describing an increased immunoreactivity of collagen IV in comparable animal models of ischemia (e.g., Hawkes et al., 2013). In contrast, Hamann and co-workers demonstrated in 1995 a decrease of collagen IV due to different durations of ischemia in the non-human primate (Hamann et al., 1995). This opposite finding is likely related to the underlying measurement technique as the number of positive vessels was used as readout, quite different from the signal intensity applied in the present study. Concerning fibronectin, the present study for the first time aims to characterize ischemic consequences in a spatio-temporal manner and thus a comparison to earlier reports is rather challenging. Referring to the few available data, Milner et al. (2008) investigated mice subjected to global hypoxia up to 14 days and reported an up-regulated fibronectin on capillaries as assessed biochemically. Huang et al. (2015) used a transient model of middle cerebral artery occlusion in mice to investigate the angiogenic response after ischemia, also including an immunofluorescence-based detection of fibronectin to count the number of positive vessels. They were found to be increased over time in the ischemic border zone (penumbra). The angiogenic response to ischemia was further investigated by Li et al. (2012) using a transient model of middle cerebral artery occlusion in mice, which led to significantly augmented fibronectin-positive vessels in the ischemic border zone.

In the present study, added quantitative analyses of immunosignals for collagen IV and fibronectin in mice revealed a significantly increased signal due to focal cerebral ischemia. With reference to regional aspects along the ischemia-affected neocortex, maximum signals were observed in the area of direct ischemia with a gradual decline toward the lesser affected ischemic border zone and the adjoined non-affected area. Significantly increased immunosignals for both collagen IV and fibronectin were observed in the ischemia-affected subcortex, i.e., the striatum. Remarkably, the ischemia-related increase in the immunoreactivities of collagen IV and fibronectin was found in a comparable manner between 4 and 24 h of ischemia, although a trend toward a numerical increase at 24 h was noted. Solely in the ischemia-affected subcortex, the immunosignal of fibronectin increased statistically over time when compared to the contralateral site.

Overall, this study identified significantly enhanced immunosignals for collagen IV and fibronectin following focal cerebral ischemia in mice, rats, sheep, and in human stroke tissue. Derived from the observed close regional association of collagen IV and fibronectin – and considering their primary locations with reference to the basement membrane and the adjoined ECM (del Zoppo and Milner, 2006; Singh et al., 2010; Gatseva et al., 2019) – this study provides robust evidence for an ischemia-induced critical affection of the NMZ. Given the fact of the assumed highly dynamic situation at this site, different patterns of the collagen IV and especially the fibronectin immunosignal would have been expected over time. Although a trend to an increasing immunoreactivity was noted toward an ischemia duration of 24 h when compared to the earlier time point of 4 h, this study revealed consistently increased immunosignals of collagen IV and fibronectin up to 3 weeks following the ischemic event as exemplarily shown by the human stroke sections.

As the present study was designed rather descriptive, a conclusion regarding the causal relationship of altered collagen IV and fibronectin immunosignals remains to be elucidated. However, some thoughts on the potential pathophysiological background might be extracted from earlier reports. Yang et al. (2007) applied a rat model of transient focal cerebral ischemia together with the application of tissue-type plasminogen activator (tPA) as a routinely used thrombolytic substance in the clinical scenario. Thereby, a decreased number of collagen IV-positive vessels were found due to ischemia, and the addition of tPA resulted in a dose-dependent decrease in the tissue content of collagen IV, fibronectin, and other markers. The authors concluded that the observed reduction of collagen IV and other basement membrane constituents reflects the tPA-mediated BBB disruption (Yang et al., 2007). This is very plausible as such BBB alterations are assumed to have a pivotal role for tPA-related bleeding complications (Ishrat et al., 2012; Zhang et al., 2012). With a special focus on fibronectin, Nicosia and co-workers identified fibronectin in cell culture experiments as a promotor for angiogenesis, whereby especially the length of microvessels was found to respond to fibronectin (Nicosia et al., 1993). Further evidence for this perspective was provided from Wang and Milner (2006) as the proliferation but not the survival of capillary endothelia cells was promoted by fibronectin in cell cultures. Consequently, based on the few available data, a role of fibronectin in angiogenesis following hypoxia appears plausible. However, the observation of the present study with an altered immunosignal as early as 4 h after the ischemic event leads to the assumption that properties of fibronectin are not limited to angiogenesis, but may also include acute damaging and long-lasting regenerative processes.

Notably, fibronectins’ functional role is still a matter of debate. As for example regarding tissue regeneration, a diversity of beliefs exists ranging from an initial beneficial effect toward an impairment of regeneration in later phases (Stoffels et al., 2013). The remaining difficulties in identifying a clear pathophysiological role of fibronectin might be related to varying properties in different tissues in addition to the brain, such as the skin and the cartilage. Further, different proportions of fibronectin exons like the extra domain A and B due to alternative splicing result in up to 20 different protein variants in human (White and Muro, 2011). In addition, the situation is complicated by the fact that for fibronectin a circulating, soluble form in the plasma has been identified, which is synthesized by hepatocytes, in addition to the cellular-associated, i.e., insoluble form as part of the ECM that is synthesized for instance by endothelial cells (To and Midwood, 2011). Interestingly, Sakai et al. (2001) reported different roles for the circulating fibronectin as mice deficient for this type of fibronectin showed larger infarct areas after a transient model of focal cerebral ischemia, while dermal wound healing was not affected. In an *in vitro* experiment, the soluble fibronectin was further found to impact the formation of fibrin with more and more fibrin matrices in case of increasing fibronectin concentrations (Ramanathan and Karuri, 2014). Circulating fibronectin is thus believed to have a pivotal role on clot formations (To and Midwood, 2011), which is supported by detected fibronectin in proteomic analyses of thrombi that were evacuated by endovascular treatment in human stroke (Muñoz et al., 2018). Additional data on circulating fibronectin recently emerged from an experimental study by Dhanesha et al. (2019): by applying a filament‐ and clot-based stroke model these authors were able to demonstrate that mice deficient for the fibronectin containing the extra domain A exhibited a decreased thrombo-inflammatory response. Such a reaction is typically linked to secondary thrombosis due to ischemia, which likely represent the key mechanism for the also perceived smaller infarct sizes and improved neurobehavioral deficits. Consistently, several inflammatory processes – as one of the major mechanisms for stroke-related tissue damage (Dirnagl et al., 1999) – were identified in an earlier study applying genetically altered mice including an alternatively spliced extra domain A (Khan et al., 2012). However, a variety of mainly *in vitro* experiments pointed out the complexity of alternatively spliced isoforms of fibronectin, each of them with distinct properties concerning chemokines and other signaling molecules (White et al., 2008).

Although several studies have focused on fibronectin that measured in the plasma of stroke patients, the currently available data does not support their use for distinguishing between ischemic and hemorrhagic stroke (Bustamante et al., 2017), nor to identify patients for recanalizing treatments (Rodríguez-Yáñez et al., 2011). Basically, a more comprehensive perspective seems reasonable understanding fibronectin as part of the brains’ ECM embedded in a complex network of cells related to the NVU. This perspective is supported by studies that identified serum markers of the NVU like GFAP, allowing insights into local processes of the ischemia-affected brain (e.g., Perry et al., 2019).

### Spatial Relationships Between Collagen IV, Fibronectin, and the Neurovascular Unit

In the present study, elements of the NVU were addressed by multiple fluorescence labeling based on a variety of antibodies, which have proved their usability in previous studies, in conjunction with collagen IV and fibronectin to explore spatial relationships. In detail, the vasculature was visualized by lectin-histochemistry, while immunolabeling revealed neurons by NeuN, oligodendrocytes by CNP, microglia by Iba, astrocytes by GFAP as well as their endfeet by AQP4.

Overall, the ischemia-induced affections of the NVU elements shown in this study largely confirmed earlier reports from the own group (e.g., Michalski et al., 2010; Härtig et al., 2017; Mages et al., 2019) and others (e.g., Mullen et al., 1992; Matsumoto et al., 2007). With a special focus on collagen IV and fibronectin, both were found without an overlapping signal for astrocytes as indicated by the complementary staining patterns with disappearing astrocytes toward the area of ischemia. At the border zone, morphological features of astrocytes were observed in good accordance to an astrocyte scar formation known for several brain injuries (Anderson et al., 2016; Dias and Göritz, 2018), which was also visible in our study after 2 weeks of focal cerebral ischemia in sheep. Such scar formations are of great interest concerning regenerative aspects. Especially, collagen IV was identified to be involved in the resulting inhibition of for example axon growth (Stichel et al., 1999; Hermanns et al., 2001). The present work revealed fibronectin in the area of ischemia that was only occasionally associated with microglia, whereas overlapping signals of fibronectin and neuronal structures were not unequivocally evident.

Notably, the present study elucidated a close regional association with vessels not only as expected for collagen IV but also for fibronectin that was consistently observed in the applied animal models and human stroke tissue. Qualitative analyses at higher magnification showed that the collagen IV immunoreactivity is encircled by the fibronectin signal in excellent accordance to the concept of the here addressed NMZ. With an additional focus on the regionally associated endothelial cells that were discussed to have a pivotal role in BBB integrity (Krueger et al., 2015), integrins as connecting elements of the endothelial layer and the adjacent basement membrane as well as ECM are of special interest (del Zoppo and Milner, 2006; Edwards and Bix, 2019). In the present study, integrin α_5_ was observed in close regional association to the vasculature together with fibronectin. Referring to the time course of BBB opening following focal cerebral ischemia, integrins associated with endothelial cells were found to be also time-dependently expressed (Huang et al., 2015). Thereby, the observed integrin *α*_5_ immunoreactivity 3 days after ischemia in the present study appears in good accordance with the described peak of integrin *α*_5_ at day 4 (Guell and Bix, 2014). With regard to functional consequences of changed integrin levels, the few available reports indicated a relationship to angiogenesis. In detail, Li et al. (2010) applied a mouse model of chronic global hypoxia and a cell culture experiment with endothelial cells to investigate integrins by immunofluorescence labeling and western blot analysis. They observed an increased integrin *α*_5_ expression on angiogenic brain endothelial cells; whereas, the spatio-temporal patterns were found to differ between the ligands associated with the receptors with a clear relevance for *α*_5_/*ß*_1_ over *α*_v_/*ß*_3_ concerning the proliferation of brain endothelial cells (Li et al., 2010). In a further study, Milner et al. (2008) used a mouse model of chronic global hypoxia and demonstrated integrin α_5_ being strongly upregulated on hypoxic capillaries. However, as specific tools targeting integrins are currently lacking, conclusions in terms of causal relationships remain impossible.

### Methodological Considerations

This study has some limitations: First, the methodological spectrum is limited to immunofluorescence labeling, which was chosen to explore related signals in both a qualitative and a quantitative manner and to allow comparative analyses at different time points after ischemia onset and diverse animal models or even autoptic human tissue. Consequently, future studies are needed to add analyses comprising protein levels from constituents of the NMZ, which might help to explore functional properties of this special site.

Second, this study is a first attempt to provide a spatio-temporal characterization at the site of the NMZ and thus focused on collagen IV and fibronectin as two representatives. Because collagens represent a large family consisting of several types, as for instance type II, III, or VI, that might exhibit different reactions in the setting of ischemia (Gatseva et al., 2019), further studies might try to cover more than the here addressed collagen IV. Furthermore, other structures associated with the NMZ like β-cadherin, located in close vicinity to integrins, and dystroglycans, related to astrocyte endfeet at the vasculatures’ abluminal side (del Zoppo and Milner, 2006), would be of interest concerning their ischemic consequences, and thus need to be addressed in future studies. Thereby, confocal laser scanning microscopy might further help to explore the regional, i.e., three-dimensional arrangement of these structures and the vasculature in more detail.

Third, quantifications were limited to the mouse model with ischemia duration of 4 and 24 h, which was due to the restricted availability of tissues, especially from other species and the human stroke case. Based on the consistent qualitative analyses that emerged from tissues of rat, sheep, and a human stroke, there are no doubts that an increase of the immunosignals from collagen IV und fibronectin due to focal cerebral ischemia would be quantifiable in these species too. As differences among species might be of relevance for the evaluation of therapeutic approaches, further confirmatory analyses including human brain tissue affected by ischemia seem reasonable. Notably, longitudinal investigations are of special interest to explore the temporal profile of increased fibronectin levels in the human brain after focal cerebral ischemia. As already gradual changes might be associated with different properties concerning the ischemic tolerance, a comparison with imaging parameters that visualize the tissue at risk could help to identify conditions that allow neuroprotective interventions.

Fourth, given the complexity of fibronectin isoforms and on the other hand, the antibodies used in this study, which are considered as pan-fibronectin markers, future efforts should include the detection of fibronectin isoforms in ischemic tissues.

### Summary and Outlook

This study for the first time provides a spatio-temporal characterization of ischemia-related alterations for collagen IV and fibronectin as components of the NMZ in various animal models and in human tissue. Because the immunosignals from collagen IV as part of the basal membrane and from fibronectin as a crucial component of the ECM increased concomitantly and in a long-term range after the ischemic event, this study robustly shows that ischemic consequences are not limited to the traditional NVU components and the ECM, but also involve the NMZ. There might be various properties of this special zone including BBB regulations, of which a significant impairment is known to cause severe complications after stroke. As not yet considered for therapeutic approaches in the setting of stroke, the NMZ qualifies for further research to explore its pathophysiological role and opportunities for pharmacological interventions.

## Data Availability Statement

The raw data supporting the conclusions of this article will be made available by the authors, without undue reservation.

## Ethics Statement

The animal study was reviewed and approved by Regierungspräsidium Leipzig as local authority (reference numbers: TVV 02/17 for mice and rats, and TVV 56/15 for sheep).

## Author Contributions

DM and WH designed the study and wrote the manuscript with considerable input from ES. Animal experiments including rodents were performed by DM and BM, whereas the experiments with sheep were carried out by HB and BN. Essential work concerning the human tissue was conducted by AH and CJ. Appropriate immunoreagents for this study were generated and provided by HM and SS. Histochemistry and imaging were conducted by WH, ES, WR and JP. Quantitative analyses and statistical calculations were done by ES, WR and DM. The final figures were generated by DM following proposals from WH. All authors contributed to the article and approved the submitted version.

### Conflict of Interest

The authors declare that the research was conducted in the absence of any commercial or financial relationships that could be construed as a potential conflict of interest.
